# Flower Species Ingredient Verification Using Orthogonal Molecular Methods

**DOI:** 10.3390/foods13121862

**Published:** 2024-06-13

**Authors:** Subramanyam Ragupathy, Arunachalam Thirugnanasambandam, Thomas Henry, Varathan Vinayagam, Ragupathy Sneha, Steven G. Newmaster

**Affiliations:** 1 Natural Health Product Research Alliance, College of Biological Sciences, University of Guelph, Guelph, ON N1G 2W1, Canada; athiru@outlook.com (A.T.); henryt@uoguelph.ca (T.H.); vinaychemist89@gmail.com (V.V.); snewmast@uoguelph.ca (S.G.N.); 2 College of Medicine, American University of Antigua, Jobberwock Beach Road, Coolidge P.O. Box W1451, Antigua; snehara@auamed.net

**Keywords:** flowers, nuclear magnetic resonance, natural health products, quality assurance

## Abstract

Flowers are gaining considerable interest among consumers as ingredients in food, beverages, cosmetics, and natural health products. The supply chain trades in multiple forms of botanicals, including fresh whole flowers, which are easier to identify than dried flowers or flowers processed as powdered or liquid extracts. There is a gap in the scientific methods available for the verification of flower species ingredients traded in the supply chains of multiple markets. The objective of this paper is to develop methods for flower species ingredient verification using two orthogonal methods. More specifically, the objectives of this study employed both (1) DNA-based molecular diagnostic methods and (2) NMR metabolite fingerprint methods in the identification of 23 common flower species ingredients. NMR data analysis reveals considerable information on the variation in metabolites present in different flower species, including color variants within species. This study provides a comprehensive comparison of two orthogonal methods for verifying flower species ingredient supply chains to ensure the highest quality products. By thoroughly analyzing the benefits and limitations of each approach, this research offers valuable insights to support quality assurance and improve consumer confidence.

## 1. Introduction

Flowers have been utilized in multiple products by many cultures for thousands of years providing nutrition, health benefits, personal beautification, and artistic flair in cuisine. In fact, the market supply chain of edible flowers has experienced considerable growth that is predicted to continue into the foreseeable future [1,2]. There are many factors responsible for the consumer demand in this market, including health benefits and general interest in having flowers displayed in foods or as ingredients in natural health products [3]. Mlcek and Rop [4] provided a fundamental review of edible flowers used in foods with more recent literature reviews in response to the increased research over the last decade [5,6,7]. The traditional use of flowers dates back thousands of years of which there are many records in the published literature [8,9]. The traditional utility of flowers to treat many health issues [10,11,12] has catalyzed more recent research efforts to explore mechanistic bioactive molecules that influence medicinal efficacy [13]. Detailed phytochemical research on flowers is extensive, including many common flower species, such as chrysanthemums [14,15,16], mustards [17], marigolds [18], locusts [19], daylilies [20], and many other flower species [6,21]. Some of this research has resulted in the discovery of novel compounds in flowers that could be developed into new pharmaceuticals [22,23]. More recently, the demand for flowers from consumers is related to factors not only limited to health benefits [24,25] but also culinary utility in foods to enhance consumer experience, such as in the presentation, taste, and aroma [26,27]. The increasing commercial value of edible flowers increases the demand for quality assurance tools that ensure safe and authentic flower ingredients [4,28]. Current quality assessment tests are based on the analysis of phytochemicals found in botanical ingredients sourced from leaves, stems, and roots with considerable research gaps with respect to flowers.

Flowers have different assemblages of metabolites than other plant parts such as leaves or roots. The most common phytochemicals in edible flowers, which are also sometimes called ‘specialized metabolites’ (SMs) [29], are grouped into five significant clusters according to Lu et al. [30]. These SM groups are composed of carotenoids, phenolics, alkaloids, nitrogen-containing compounds, and organosulfur compounds. In some cultures, flower color is an important indicator for specific health benefits [31]. Red and purple flowers are dietary sources of anthocyanin [32]; depending on the pH and their structural characteristics, these water-soluble compounds give off distinctive red, purple, or blue colors [33,34,35]. The mineral content of flowers is considerable in terms of both macronutrients (phosphorus, potassium, calcium, and magnesium) and micronutrients (iron, manganese, copper, and zinc) [12]. For instance, flowers from the botanical genera *Chrysanthemum*, *Dianthus*, and *Viola* are notably rich in these nutrients, particularly potassium [36]. Some *Monarda* species are also very high in calcium and magnesium [37]. Zinc, which is notably present in *Tagetes patula* flowers [36], is of nutritional significance since it plays a significant role in modifying immunological function [38]. African marigold contains phenolics, which are antioxidants that are thought to prevent chronic diseases and reduce the risk of cancer [39,40]. The presence of vitamins A, C, and E in flower tissues, such as in *Rosa hybrida*, *Borago officinalis*, *Camellia japonica*, *Centaurea cyanus*, and *Viola* × *wittrockiana*, is also a factor related to antioxidant activity and other potential health benefits [41,42]. The aromatic chemicals that are responsible for the fragrance of flowers are unique and only found in the flowers. The cyclic terpenoid β-ionone is an example of a fragrant element found in certain flowers, including *Viola*, *Petunia*, and *Boronia* [43,44,45], which exhibits an inhibitory effect on the proliferation of cancerous cells by preventing the apoptosis of human gastric adenocarcinoma cells [46,47]. Given that flowers are composed of many compounds of which some are specific and others are shared among species, it is very difficult to use a targeted analytical chemistry approach for flower species ingredient identification. Furthermore, any targeted method developed for leaves will likely not work for flowers because the targeted metabolites produced in leaves are different than those in flowers. Novel identification methods are required for the identification of flowers, particularly processed flower extracts.

Molecular diagnostic tools provide alternative methods for the identification of botanical ingredients, particularly processed ingredients. Processed ingredients no longer have morphological characters required for classical taxonomic identification. Molecular methods for botanical identification include the use of both DNA-based methods and the analysis of phytochemicals present within botanical ingredients. DNA-based tools have been used for botanical ingredient identification to verify species ingredients on labels and unwanted adulterant species [48,49,50]. Targeted DNA methods have been validated and utilized within the food, herbal, and supplement industries [51]. These DNA-based tools have been used successfully by these industries to verify plant species in sourced ingredients when samples have a sufficient quantity of good-quality DNA [52,53]. Although there is considerable research published on DNA-based identification and authentication that utilize leaves, fruit/seeds, and roots, there are only a few published studies that have used DNA methods on flowers to identify plant species [54,55].

Alternative methods, such as the use of analytical chemistry, has been shown to be very useful in the identification of botanical ingredients [56]. In fact, the use of targeted analytical chemistry methods has become an industry standard for verifying specific phytochemicals that are present in botanical ingredients [57]. However, there are considerable limitations to the use of targeted analytical chemistry for species identity testing due to a limited number of plant species having known specific metabolites that can be easily identified [57,58,59]. Non-targeted methods such as proton nuclear magnetic resonance (^1^H NMR) can non-destructively identify a wide range of phytochemicals present in botanical ingredients [60,61]. Considered as a plant metabolite fingerprint, ^1^H NMR provides spectra in which the areas of signal peaks are proportional to the total number of protons and relative concentrations in samples providing a qualitative and quantitative analysis of phytochemicals in a sample [62]. This method has been used for the identification of botanicals in natural herbals [63,64,65,66,67], meat identification and adulteration [68,69,70,71], fraudulent dairy food [72], fish fraud and mislabeling [73,74], coffee authentication and blend quantification [75], and quality control of wine [76]. Plants produce different metabolites within different plant parts, such as flowers, leaves, stems and roots, and these metabolite differences can be used to differentiate plant parts using NMR metabolite fingerprints [77]. NMR profiling techniques can also be used to identify different flower color varieties, as was recorded for four different flower varieties of *Catharanthus roseus* plants [78]. However, to our knowledge, there is no published literature on the use of ^1^H NMR to identify flower species or flowers used as ingredients in food or natural health products.

The objective of this paper is to develop methods for the identification of flower parts for botanical species ingredient verification using two orthogonal methods. We collected flowers from 33 samples representing 23 botanical species, which were identified using traditional taxonomic morphological methods with the preparation and archival of reference material herbarium vouchers. More specifically, the objectives of this study employed both (1) DNA-based molecular diagnostic methods and (2) NMR metabolite fingerprint methods in the identification of 23 common flower species ingredients used in food, beverage, and natural health products. This study provides a comparison of the benefits and limitations of alternative methods for flower species ingredient supply chain verification needed to support quality assurance in the food, beverage, and natural health product industries.

## 2. Materials and Methods

A total of 33 flower samples representing 23 species were gathered from the University of Guelph Arboretum, local parks, flower markets, and other locations around Guelph, Ontario, Canada. These 33 samples were identified using morphological floristic and vegetative characteristics and traditional taxonomic methods of identification (Table S1). Only two samples (*Hibiscus sabdariffa*; *Sphaeranthus indicus*) were air-dried at room temperature for one week and stored at room temperature. All other samples were collected fresh and stored at 4 degrees Celsius for 48 h. All flowers were pulverized, immediately followed by NMR extraction protocols defined below. Additionally, DNA was directly extracted from the fresh leaves and flowers. Flowers were sampled mid-morning (8–10 AM), packaged in plastic bags, and placed in refrigeration (4 °C) by noon time. Flowers were cleansed with distilled water; then, the petals were removed and left to drip at room temperature. Herbarium vouchers were created for each specimen and deposited in the College of Biological Science (CBS), Natural Health Products Research Alliance (NHPRA) OAC Herbarium at the University of Guelph.

Genomic DNA from flower and leaf samples was extracted using a Nucleospin Plant II kit (Macherey-Nagel GmbH & Co. KG, Düren, Germany) to obtain high-quality DNA. Extractions were performed using 100 mg of each sample according to the manufacturer’s instructions. DNA quantification was performed using the Qubit^TM^ 3.0 Fluorometer (Life Technologies Holdings, Marsiling Industrial Estate Road, Singapore). For each species, four regions were sequenced: the plastid regions, *rbcL* and *trnH-psbA*; the nuclear region, *ITS2*; and a species-specific mini barcode marker designed for each species in this study using the IDT PrimerQuest™ (Integrated DNA Technologies, Inc., Coralville, IA, USA) Tool (see Table S2 for a list of primers used). The PCR was performed under standard conditions for the primer pairs as described in Fazekas et al. [79] (see Table S2 for the annealing temperatures used for each primer and also see [80,81,82,83,84,85,86,87]). PCR products were bidirectionally sequenced using BigDye™ (Thermo Fisher Scientific, Vilnius, Lithuania) sequencing reactions, and the sequence products were analyzed on an ABI 3730 DNA Analyzer (Thermo Fisher Scientific, Waltham, Massachusetts, United States) at the University of Guelph Genomics Facility. Chromatographic traces were edited and assembled into contiguous alignments using CodonCode Aligner (version 10.0.2). Sequence identity was checked against the GenBank and BOLD databases.

The methods of extraction and NMR analysis were refined from NMR methods developed in earlier publications [65,66,75,88].

For methanol extraction, a flower sample of 300 mg of fresh weight was transferred to a 15 mL glass vial. Then, 2 mL of a methanol solution containing 10% deuterated methanol and 0.05% TMS (tetramethylsilane) was added to the vial. The purpose of using 10% deuterated methanol was to lock the proton during the proton NMR data collection and reduce the cost of the solvent as much as possible. Sample vials were tightly closed with a polypropylene screw cap and kept in a water bath for 2 h at 60 °C. All sample decoctions were vortexed for 2 min, placed in a sonicating bath (40 Hz) for 5 min, and centrifuged for 15 min at 6000 rpm at room temperature. The clear supernatant (650 μL) was transferred to a 5 mm NMR tube for the ^1^H-NMR analysis.

For water extraction, 300 mg of fresh flower sample was weighed and transferred to a 15 mL glass vial, and 2 mL of 9:1 water solution (H_2_O:D_2_O with 0.01% TSP (trimethylsilyl propionate)) was added. Tightly closed sample vials were kept in a water bath for 2 h at 80 °C, vortexed for 2 min, placed in a sonicating bath (40 Hz) for 5 min, and centrifuged for 15 min at 6000 rpm at room temperature. The supernatant was collected in a 5 mm NMR tube for ^1^H-NMR spectral acquisition. All solvents and internal standards were purchased from Sigma-Aldrich, Oakville, ON, Canada.

One-dimensional proton NMR NOESY spectra were acquired on the 400 MHz Bruker AVANCE III spectrometer (Bruker Switzerland AG, Fallanden, Switzerland), using Bruker’s standard NOESY pulse program with water and methanol suppression. To acquire high-quality spectra, 15 s acquisition delay, 3.988 sec acquisition time, 2.54 ppm spectral width, 16 receiver gain, and 64 scans were performed on every sample. The data were analyzed using Bruker Topspin 4.0.7 with a resolution of 32K data points. Peak assignment and organic molecule identification were performed using MestReNova (version 14.0.0). Spectra for 33 flower samples were acquired based on the resonance signals from the nature of protons of metabolites present in the flower samples. Variation among the NMR signal positions and amplitudes indicates the variation in the metabolite content and concentration among the 33 flower samples. ^1^H-NMR spectra were processed using TopSpin 4.0.7. The phase and baseline were checked and corrected to ensure all spectra were of high quality. Spectra were calibrated to the TMS/TSP peak at 0.0 ppm. Processed spectra were bucketed with simple rectangular buckets of positive intensities without scaling (AMIX 4.0.1). The chemical range utilized for bucketing was 1 to 12 ppm, with a width of 0.01 ppm. While bucketing, the residual solvent signals of water, methanol, and TMSP were removed at the regions 4.75 to 5.06, 3.16 to 3.45, and -0.05 to 0.05 ppm, respectively. After bucketing, each spectrum was normalized by setting the below means as 0, and the above means were binned from 1 to 100. The multivariate statistical method (Hierarchical Cluster Analysis) was used to analyze NMR spectra [65,66] and classify flower species based on the similarity of metabolite fingerprint spectra. Hierarchical cluster analysis (HCA) was performed using the Euclidean distance matrix with Ward’s linkage algorithm.

## 3. Results

**Figure 1:**
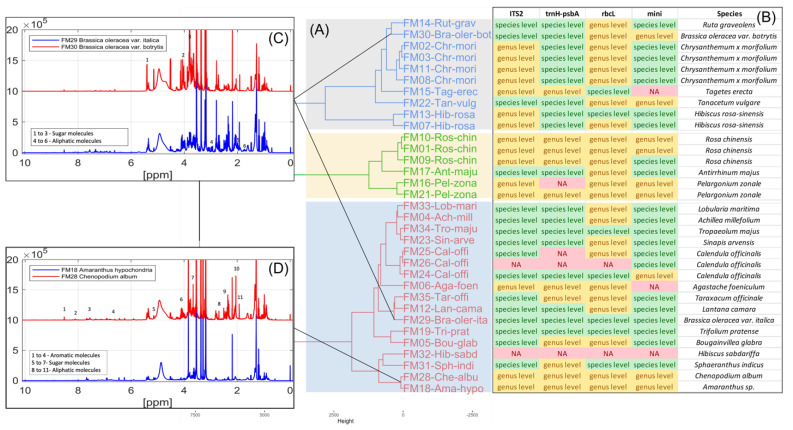
Classification of variation in flower metabolites from 33 flower samples representing 23 species, including DNA-based species identification. (**A**) NMR metabolomic dendrogram. (**B**) Identification of flowers using four different DNA regions. Inset (**C**) NMR spectra representing the large differences in metabolites among distant groups in the dendrogram; different varieties of *Brassica oleracea* have very different plant metabolites. (**D**) NMR spectra representing the small differences in metabolites within near groups in the dendrogram.

The orthogonal analysis of the flower samples resulted in differences in taxonomic resolution based on the DNA sequence and NMR spectral analyses. DNA methods using any of the four different primer sets resulted in a lower species/variety resolution than the NMR methods. Combining all four primer sets, DNA species resolution was 17/23 species but had no resolution of flower varieties and failures for PCR or sequencing for several samples (Table 1). The NMR spectral fingerprint analysis of variation in metabolites clearly differentiated all 23 species, including varieties and flower color variants (Figure 1).

**Figure 2:**
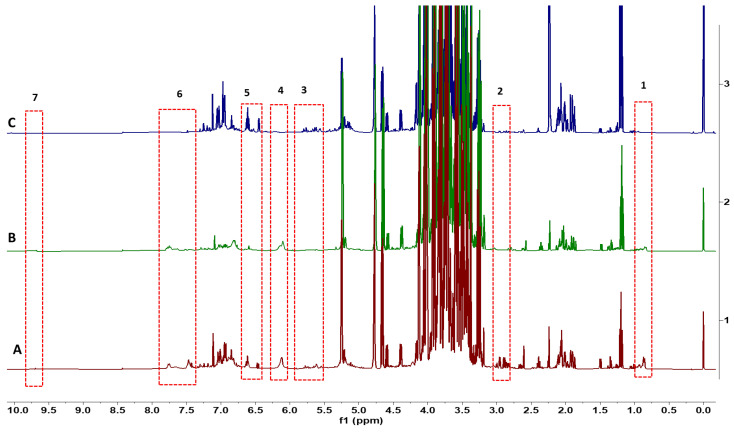
A stacked comparison ^1^H-NMR spectra of three different colored flowers of *Rosa chinensis*: (**A**) *Rosa chinensis*—pink flower; (**B**) *Rosa chinensis*—white with pink spotted flower; (**C**) *Rosa chinensis*—red flower. Numbered boxes represent differentiating peaks among samples/taxa.

**Figure 3:**
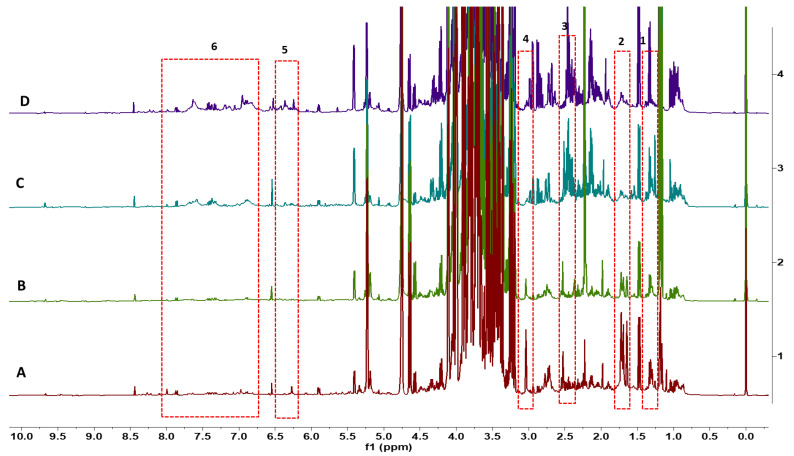
A comparison of the stacked ^1^H NMR spectra of extracts from edible flowers: (**A**) *Chrysanthemum* × *morifolium*—white flowers; (**B**) *Chrysanthemum* × *morifolium*—yellow flowers; (**C**) *Chrysanthemum* × *morifolium*—yellow flowers; (**D**) *Chrysanthemum* × *morifolium*—yellow-orange flowers. Numbered boxes represent differentiating peaks among samples/taxa.

**Figure 4:**
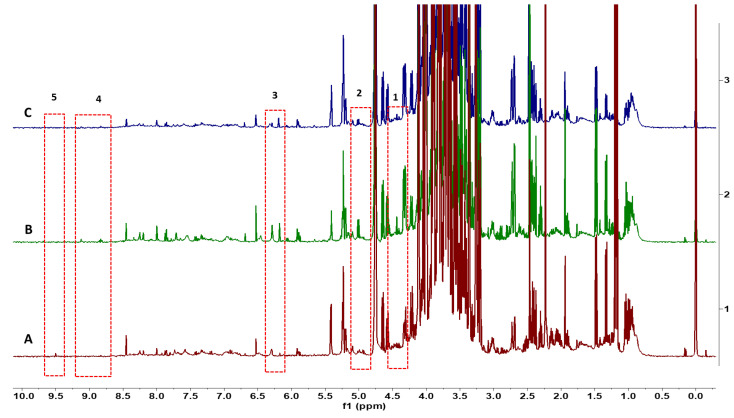
A comparison of the ^1^H-NMR spectra of different colors *Calendula officinalis* flowers: (**A**) *Calendula officinalis*—orange flowers; (**B**) *Calendula officinalis*—yellow flowers; (**C**) *Calendula officinalis*—orange-yellow flowers. Numbered boxes represent differentiating peaks among samples/taxa.

**Figure 5:**
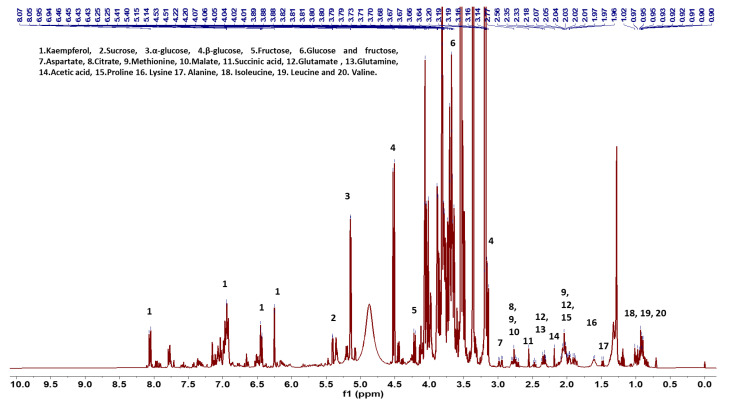
^1^H-NMR spectra of extracts of flowers with the assigned metabolites.

NMR spectra could be used to classify all flower species. The variation in spectra provides a classification of all 23 flower species based on the metabolites found in the flowers of each species. Multiple flowers per species confirmed consistency in the metabolite spectra within a species. The dendrogram (Figure 1) identifies clusters of flower species that have similar metabolites. Distant groups of taxa (Figure 1A) have NMR spectra representing the large differences in metabolites among species from groups that are further away in the dendrogram. For example, different varieties of *Brassica oleracea* have very different plant metabolites, including sugars and aliphatic molecules unique to each variety of *Brassica oleracea*. Flower species with similar NMR spectra have relatively fewer differences in metabolites within near groups in the dendrogram (Figure 1B). Although the spectra for flowers from *Chenopodium album* and *Amaranthus hypochondriacus* are similar for these small-flowered plants, there are differences in the metabolites, including some aromatic molecules. This is the first study to record metabolite spectra for flowers that may be useful as an identification tool for flowers. There were differences in the spectra of three different colored flowers of *Rosa chinensis*, including pink flowers, white flowers with pink spots, and red flowers (Figure 2). The spectral differences among the three different colors of *Chrysanthemum* × *morifolium* flowers were compared, and the differences are shown in Figure 3; the peaks represent aliphatic acids and amino acids (Figure 3: red boxes 1–3), and aromatic molecules are presented such as flavonoids and anthocyanins (Figure 3: red boxes 4–5). Similar results were observed in *Calendula officinalis*, which has three different flower color variants that correspond with the variation in the spectra. An additional peak is observed in yellow and orange-yellow flowers, corresponding to carbohydrates (Figure 4: boxes 1 and 2). The flavonoid peaks are observed in two color variants (Figure 4B,C: box 4). The peak representing alkenes (box 3 in Figure 4B,C) is not seen in the orange variant (Figure 4A). The orange (Figure 4A) color in these flowers also has an aromatic aldehyde/acids peak that is not observed in other color flowers (Figure 4B,C). We could identify 20 known compounds using a ^1^H-NMR spectrum (Figure 5), including different (low to higher) molecular masses. Among all spectra, the general signal distribution properties in the region of δ 10.00–0.00. A typical ^1^H NMR spectrum can be divided into three regions: the aromatic region (δ 10.00–6.00), the sugar region (δ 6.00–3.50), and the organic and amino acid region (δ 3.50–0.00). In the aromatic compound region (δ 10.00–6.00), flavonoids and phenolic acids were identified. In the middle part of the sugar region (δ 6.00–3.0), sugar, glucose, and fructose were identified as the significant sugars. In the organic acid region (δ 3.0–0.0), aspartate, citrate methionine, malate, succinic acid, glutamate, glutamine, acetic acid, proline, lysine, alanine, isoleucine, leucine, and alanine were identified. All the metabolites assigned are listed in Table 2.

## 4. Discussion

The DNA methods resulted in a taxonomic resolution that was limited by several factors. Firstly, there is a variation in the taxonomic resolution among the four regions (Figure 1). Combining all four markers, 17 total species could be identified, with the greatest resolution obtained using the species-specific mini barcode primers (16 species), followed by the *ITS2* (15 species), *trnH-psbA* (14 species), and *rbcL* (7 species) regions, respectively. This is not a surprising result as several papers have compared the species resolution of these genetic regions and found similar patterns of variation in species resolution for these DNA regions [79,86,89]. The higher success of the mini barcode primers was expected as they target DNA regions specifically selected to maximize species resolution, and species-specific primers can have higher PCR affinity and better amplification success vs. universal plant barcode primers. The shorter mini barcodes can yield also higher sequencing success with degraded and fragmented DNA [90]. Sequencing was successful for 22/23 species with failure in only one species (*Hibiscus sabdariffa*) of which we could not obtain sequences for any of the regions tested. Previous research has discussed issues in resolving related species of *Hibiscus* from *Hibiscus sabdariffa* for various DNA regions and suggested the region *matK*, which was not employed in our study [91]. Although both leaves and flowers were used to identify the same 17 species, there was much greater sequencing success and species resolution using leaf DNA (Table 1). Further developments of the methods of extraction for flowers are needed as the quantity and quality of isolated genomic is thought to be lower in petal tissues than leaf tissues [92,93]. The utility of DNA methods to identify species using flowers may be improved with better extraction methods while using a tiered approach [94] with *trnH-psbA*, *ITS2*, and mini barcode regions.

This study provides a comparison of two methods that could be used for the identification of flowers. DNA methods can be used for flowers that have adequate amounts of high-quality DNA, but this can sometimes be a challenge for processed flower samples. NMR metabolite analysis was useful for the identification of all flower taxa in this study. These metabolite fingerprints may be useful for identifying flowers used in ingredients supporting quality assurance programs. Additionally, ^1^H-NMR can be used for the quantitative and qualitative analysis of metabolites thought to be bioactive and the basis for efficacy and label claims in natural health products. We used NMR technology to identify some specific plant compounds. This allowed several primary and secondary metabolites to be identified based on information from previous publications on ^1^H-NMR [95,96]. Further research is needed to document metabolite variation among flowers for botanical species that are used commercially in natural health products to support the quality control of flower products. These advances form a basis for breeding programs and understanding basic phytochemical mechanisms that underpin efficacy observed in clinical trials. This structural metabolomic approach founded on quantitative NMR could be linked to clinical evidence, thus providing the basis for further experimental research on metabolite dose in natural products.

Our research provides a method for quality assurance testing to ensure products are authentic, safe, and meet quality standards from harvesting to processing and storage. Creating a library of specific metabolite fingerprints for flower species can ensure the authenticity of sourced flower ingredients. There are safety issues concerning flower ingredients. including contamination with anti-nutritional compounds, toxins from the environment, harmful bacteria, or phytochemicals [97,98,99,100]. Metabolite fingerprints can also be used to screen for contaminants, such as external impurities occurring in edible flowers, including bacteria and chemical compounds, which alter pure metabolite spectra from specific flower species.

The need for quality standards requires further investigation on the impacts of agronomy and processing on flower metabolites [21]. Flower metabolite libraries could be developed for specific geographic regions and agricultural practices [101] based on similar research for commodities such as coffee [102,103], chocolate [104,105], and olive oil [106,107]. This includes the consideration of flower storage [108] as some of the current research in our lab suggests there may be impacts on flower metabolites after different storage treatments. The NMR metabolite analysis used in our study provides a method for investigating preservation methods [109] that may extend the shelf life of flower ingredients. Improvements in quality standards can be underpinned by methods such as the metabolite analysis used in this study, which enable the potential benefits of consuming pure, authentic flowers.

## Figures and Tables

**Table 1:** Sequence success and species resolution for four DNA regions from 33 leaf and flower samples representing 23 species.

		*rbcL*	*trnH-psbA*	*ITS2*	Mini Barcode
Sequence Success	Leaf	94	88	94	91
(% of 33 total samples)	Flower	30	39	79	73
Species Resolution	Leaf	30	61	65	70
(% of 23 total species)	Flower	13	44	61	61

**Table 2:** Chemical shifts for metabolites identified in the ^1^H NMR spectra of a methanol extract.

S. No.	Metabolites	δ ^1^H (Multiplicity)	Assignment
1	Kaempferol	8.07 (d, *J* = 8.9), 6.96 (d, *J* = 3.4),6.44 (dd, *J* = 2.0, 2.1)	Aromatic—2,6-CH
2	Sucrose	5.41 (d, *J* = 3.8)	Furanose—CH
3	α-glucose	5.15 (d, *J* = 3.6)	*α*-anomeric CH
4	β-glucose	4.53 (d, *J* = 7.8)	*β*-anomeric CH
5	Fructose	4.22 (d, *J* = 7.8)	Furanose—CH
6	Glucose and Fructose	3.89–3.49 (m)	CH, CH_2_
7	Aspartate	2.99 (dd, *J* = 3.4, 3.5)	*β*-CH_2_
8	Citrate	2.79 (dd, *J* = 5.6, 7.5)	*α*-CH_2_
9	Methionine	2.77 (m)	CH_2_
10	Malate	2.73 (m)	*α*-CH_2_
11	Succinic acid	2.56 (s)	CH_2_
12	Glutamate	2.37 (m), 2.02 (m)	*α*-CH_2_, *β*-CH_2_
13	Glutamine	2.32 (m)	CH_2_
14	Acetic acid	2.18 (s)	CH_3_
15	Proline	1.91 (m)	*β*-CH_2_
16	Lysine	1.61 (m)	CH_2_
17	Alanine	1.49 (d, *J* = 7.2)	*β*-CH_3_
18	Isoleucine	3.66 (m), 1.96 (m), 1.47 (m), 1.26 (m), 1.01 (d, *J* = 2.0), 0.96 (t, *J* = 2.3)	CH_2_, -CH3
19	Leucine	3.72 (m), 1.67 (m), 0.95 (m)	*β*-CH_2_, -CH
20	Valine	2.26 (m), 1.06 (d, *J* = 7), 0.91 (d, *J* = 3.6)	*α*-CH, *β*-CH_3_

Abbreviation: s = singlet, d = doublet, dd = doublet of doublet, m = multiplet, t = triplet.

## Data Availability

DNA sequences generated for this project were uploaded to Genbank (accession numbers are listed in Table S1). Primer sequences for PCR primers use are available in Table S2. NMR data are available from corresponding authors.

## Supplementary Materials

The following supporting information can be downloaded at: https://www.mdpi.com/article/10.3390/foods13121862/s1, Table S1: Sample list and Genbank accession IDs; Table S2: List of PCR primers used.
