# Supplementary material for: Flower Species Ingredient Verification Using Orthogonal Molecular Methods
Source: Foods. 2024 Jun 13;13(12):1862. doi: 10.3390/foods13121862 (PMC11203286; doi:10.3390/foods13121862)
Supplement: Supplementary file 1 [file foods-13-01862-s001.zip › foods-3029367-supplementary.pdf]

Table S1. Sample list and Genbank accession IDs.

Sample	Species	Tissue	Primer	Genbank ID
FM1	*Rosa sp.*	Leaf	*rbcL*	OR381714
FM1	*Rosa sp.*	Leaf	*psbA-trnH*	OR381751
FM1	*Rosa sp.*	Leaf	*ITS2*	OR400863
FM1	*Rosa sp.*	Leaf	Mini	OR400872
FM2	*Chrysanthemum x morifolium*	Leaf	*rbcL*	OR381703
FM2	*Chrysanthemum x morifolium*	Leaf	*psbA-trnH*	OR381740
FM2	*Chrysanthemum x morifolium*	Leaf	*ITS2*	OR400817
FM2	*Chrysanthemum x morifolium*	Flower	*ITS2*	OR400821
FM2	*Chrysanthemum x morifolium*	Leaf	Mini	OR381770
FM2	*Chrysanthemum x morifolium*	Flower	Mini	OR381774
FM3	*Chrysanthemum x morifolium*	Leaf	*rbcL*	OR381704
FM3	*Chrysanthemum x morifolium*	Leaf	*psbA-trnH*	OR381741
FM3	*Chrysanthemum x morifolium*	Leaf	*ITS2*	OR400818
FM3	*Chrysanthemum x morifolium*	Flower	*ITS2*	OR400822
FM3	*Chrysanthemum x morifolium*	Leaf	Mini	OR381771
FM3	*Chrysanthemum x morifolium*	Flower	Mini	OR381775
FM4	*Achillea millefolium*	Leaf	*rbcL*	OR381687
FM4	*Achillea millefolium*	Flower	*rbcL*	OR381688
FM4	*Achillea millefolium*	Leaf	*psbA-trnH*	OR381727
FM4	*Achillea millefolium*	Flower	*psbA-trnH*	OR381728
FM4	*Achillea millefolium*	Leaf	*ITS2*	OR400797
FM4	*Achillea millefolium*	Flower	*ITS2*	OR400798
FM4	*Achillea millefolium*	Leaf	Mini	OR400861
FM4	*Achillea millefolium*	Flower	Mini	OR400862
FM5	*Bougainvillea glabra*	Leaf	*rbcL*	OR381695
FM5	*Bougainvillea glabra*	Leaf	*psbA-trnH*	OR381733
FM5	*Bougainvillea glabra*	Leaf	*ITS2*	OR400805
FM5	*Bougainvillea glabra*	Flower	*ITS2*	OR400806
FM5	*Bougainvillea glabra*	Leaf	Mini	OR400867
FM5	*Bougainvillea glabra*	Flower	Mini	OR400868
FM6	*Agastache sp.*	Leaf	*rbcL*	OR381689
FM6	*Agastache sp.*	Flower	*rbcL*	OR381690
FM6	*Agastache sp.*	Leaf	*psbA-trnH*	OR381729
FM6	*Agastache sp.*	Flower	*psbA-trnH*	OR381730
FM6	*Agastache sp.*	Leaf	*ITS2*	OR400799
FM6	*Agastache sp.*	Flower	*ITS2*	OR400800
FM6	*Agastache sp.*	Flower	Mini	OR400849
FM7	*Hibiscus rosa-sinensis*	Leaf	*rbcL*	OR381707
FM7	*Hibiscus rosa-sinensis*	Flower	*rbcL*	OR381708
FM7	*Hibiscus rosa-sinensis*	Leaf	*psbA-trnH*	OR381744
FM7	*Hibiscus rosa-sinensis*	Flower	*psbA-trnH*	OR381745
FM7	*Hibiscus rosa-sinensis*	Leaf	*ITS2*	OR400825
FM7	*Hibiscus rosa-sinensis*	Flower	*ITS2*	OR400827
FM7	*Hibiscus rosa-sinensis*	Leaf	Mini	OR381778
FM7	*Hibiscus rosa-sinensis*	Flower	Mini	OR381779
FM8	*Chrysanthemum x morifolium*	Leaf	*rbcL*	OR381705

FM8	*Chrysanthemum x morifolium*	Leaf	*psbA-trnH*	OR381742
FM8	*Chrysanthemum x morifolium*	Leaf	*ITS2*	OR400819
FM8	*Chrysanthemum x morifolium*	Flower	*ITS2*	OR400823
FM8	*Chrysanthemum x morifolium*	Leaf	Mini	OR381772
FM8	*Chrysanthemum x morifolium*	Flower	Mini	OR381776
FM9	*Rosa sp.*	Leaf	*rbcL*	OR381716
FM9	*Rosa sp.*	Leaf	*psbA-trnH*	OR381753
FM9	*Rosa sp.*	Leaf	*ITS2*	OR400834
FM9	*Rosa sp.*	Leaf	Mini	OR400856
FM9	*Rosa sp.*	Flower	Mini	OR400858
FM10	*Rosa sp.*	Leaf	*rbcL*	OR381715
FM10	*Rosa sp.*	Leaf	*psbA-trnH*	OR381752
FM10	*Rosa sp.*	Leaf	*ITS2*	OR400864
FM10	*Rosa sp.*	Leaf	Mini	OR400873
FM10	*Rosa sp.*	Flower	Mini	OR400857
FM11	*Chrysanthemum x morifolium*	Leaf	*rbcL*	OR381702
FM11	*Chrysanthemum x morifolium*	Leaf	*psbA-trnH*	OR381739
FM11	*Chrysanthemum x morifolium*	Leaf	*ITS2*	OR400816
FM11	*Chrysanthemum x morifolium*	Flower	*ITS2*	OR400820
FM11	*Chrysanthemum x morifolium*	Leaf	Mini	OR381769
FM11	*Chrysanthemum x morifolium*	Flower	Mini	OR381773
FM12	*Lantana camara*	Leaf	*rbcL*	OR381709
FM12	*Lantana camara*	Flower	*rbcL*	OR381710
FM12	*Lantana camara*	Leaf	*psbA-trnH*	OR381746
FM12	*Lantana camara*	Flower	*psbA-trnH*	OR381747
FM12	*Lantana camara*	Leaf	*ITS2*	OR400828
FM12	*Lantana camara*	Flower	*ITS2*	OR400829
FM12	*Lantana camara*	Leaf	Mini	OR400854
FM12	*Lantana camara*	Flower	Mini	OR400855
FM13	*Hibiscus rosa-sinensis*	Leaf	*rbcL*	OR381706
FM13	*Hibiscus rosa-sinensis*	Leaf	*psbA-trnH*	OR381743
FM13	*Hibiscus rosa-sinensis*	Leaf	*ITS2*	OR400824
FM13	*Hibiscus rosa-sinensis*	Flower	*ITS2*	OR400826
FM13	*Hibiscus rosa-sinensis*	Leaf	Mini	OR381777
FM14	*Ruta graveolens*	Leaf	*rbcL*	OR381717
FM14	*Ruta graveolens*	Leaf	*psbA-trnH*	OR381754
FM14	*Ruta graveolens*	Flower	*psbA-trnH*	OR381755
FM14	*Ruta graveolens*	Leaf	*ITS2*	OR400835
FM14	*Ruta graveolens*	Flower	*ITS2*	OR400836
FM14	*Ruta graveolens*	Leaf	Mini	OR400878
FM14	*Ruta graveolens*	Flower	Mini	OR400879
FM15	*Tagetes erecta*	Leaf	*rbcL*	OR381720
FM15	*Tagetes erecta*	Leaf	*psbA-trnH*	OR381759
FM15	*Tagetes erecta*	Flower	*psbA-trnH*	OR381760
FM15	*Tagetes erecta*	Leaf	*ITS2*	OR400840
FM15	*Tagetes erecta*	Flower	*ITS2*	OR400841
FM15	*Tagetes erecta*	Flower	Mini	OR400874
FM16	*Pelargonium zonale*	Leaf	*rbcL*	OR381712
FM16	*Pelargonium zonale*	Leaf	*ITS2*	OR400832

FM16	*Pelargonium zonale*	Leaf	Mini	OR400870
FM17	*Antirrhinum majus*	Leaf	*rbcL*	OR381693
FM17	*Antirrhinum majus*	Flower	*rbcL*	OR381694
FM17	*Antirrhinum majus*	Leaf	*psbA-trnH*	OR381732
FM17	*Antirrhinum majus*	Leaf	*ITS2*	OR400803
FM17	*Antirrhinum majus*	Flower	*ITS2*	OR400804
FM17	*Antirrhinum majus*	Leaf	Mini	OR400850
FM17	*Antirrhinum majus*	Flower	Mini	OR400851
FM18	*Amaranthus sp.*	Leaf	*rbcL*	OR381691
FM18	*Amaranthus sp.*	Flower	*rbcL*	OR381692
FM18	*Amaranthus sp.*	Leaf	*psbA-trnH*	OR381731
FM18	*Amaranthus sp.*	Leaf	*ITS2*	OR400801
FM18	*Amaranthus sp.*	Flower	*ITS2*	OR400802
FM18	*Amaranthus sp.*	Leaf	Mini	OR400865
FM18	*Amaranthus sp.*	Flower	Mini	OR400866
FM19	*Trifolium pratense*	Leaf	*rbcL*	OR381724
FM19	*Trifolium pratense*	Flower	*rbcL*	OR381725
FM19	*Trifolium pratense*	Leaf	*psbA-trnH*	OR381764
FM19	*Trifolium pratense*	Flower	*psbA-trnH*	OR381765
FM19	*Trifolium pratense*	Leaf	*ITS2*	OR400845
FM19	*Trifolium pratense*	Flower	*ITS2*	OR400846
FM19	*Trifolium pratense*	Leaf	Mini	OR400859
FM19	*Trifolium pratense*	Flower	Mini	OR400860
FM21	*Pelargonium zonale*	Leaf	*rbcL*	OR381713
FM21	*Pelargonium zonale*	Leaf	*psbA-trnH*	OR381750
FM21	*Pelargonium zonale*	Leaf	*ITS2*	OR400833
FM21	*Pelargonium zonale*	Leaf	Mini	OR400871
FM22	*Tanacetum vulgare*	Leaf	*rbcL*	OR381721
FM22	*Tanacetum vulgare*	Flower	*rbcL*	OR381722
FM22	*Tanacetum vulgare*	Leaf	*psbA-trnH*	OR381761
FM22	*Tanacetum vulgare*	Flower	*psbA-trnH*	OR381762
FM22	*Tanacetum vulgare*	Leaf	*ITS2*	OR400842
FM22	*Tanacetum vulgare*	Flower	*ITS2*	OR400843
FM22	*Tanacetum vulgare*	Leaf	Mini	OR381780
FM22	*Tanacetum vulgare*	Flower	Mini	OR381781
FM23	*Sinapis arvensis*	Leaf	*rbcL*	OR381718
FM23	*Sinapis arvensis*	Leaf	*psbA-trnH*	OR381756
FM23	*Sinapis arvensis*	Flower	*psbA-trnH*	OR381757
FM23	*Sinapis arvensis*	Leaf	*ITS2*	OR400837
FM23	*Sinapis arvensis*	Flower	*ITS2*	OR400838
FM23	*Sinapis arvensis*	Leaf	Mini	OR400886
FM24	*Calendula officinalis*	Leaf	*rbcL*	OR381698
FM24	*Calendula officinalis*	Leaf	*psbA-trnH*	OR381736
FM24	*Calendula officinalis*	Leaf	*ITS2*	OR400809
FM24	*Calendula officinalis*	Flower	*ITS2*	OR400811
FM24	*Calendula officinalis*	Leaf	Mini	OR400880
FM24	*Calendula officinalis*	Flower	Mini	OR400883
FM25	*Calendula officinalis*	Leaf	*rbcL*	OR381699
FM25	*Calendula officinalis*	Leaf	*ITS2*	OR400810

FM25	*Calendula officinalis*	Flower	*ITS2*	OR400812
FM25	*Calendula officinalis*	Leaf	Mini	OR400881
FM25	*Calendula officinalis*	Flower	Mini	OR400884
FM26	*Calendula officinalis*	Flower	*ITS2*	OR400813
FM26	*Calendula officinalis*	Leaf	Mini	OR400882
FM26	*Calendula officinalis*	Flower	Mini	OR400885
FM28	*Chenopodium album*	Leaf	*rbcL*	OR381700
FM28	*Chenopodium album*	Flower	*rbcL*	OR381701
FM28	*Chenopodium album*	Leaf	*psbA-trnH*	OR381737
FM28	*Chenopodium album*	Flower	*psbA-trnH*	OR381738
FM28	*Chenopodium album*	Leaf	*ITS2*	OR400814
FM28	*Chenopodium album*	Flower	*ITS2*	OR400815
FM28	*Chenopodium album*	Leaf	Mini	OR381767
FM28	*Chenopodium album*	Flower	Mini	OR381768
FM29	*Brassica oleracea var. italica*	Flower	*rbcL*	OR381696
FM29	*Brassica oleracea var. italica*	Flower	*psbA-trnH*	OR381734
FM29	*Brassica oleracea var. italica*	Flower	*ITS2*	OR400807
FM29	*Brassica oleracea var. italica*	Flower	Mini	OR400852
FM30	*Brassica oleracea var. botrytis*	Flower	*rbcL*	OR381697
FM30	*Brassica oleracea var. botrytis*	Flower	*psbA-trnH*	OR381735
FM30	*Brassica oleracea var. botrytis*	Flower	*ITS2*	OR400808
FM30	*Brassica oleracea var. botrytis*	Flower	Mini	OR400853
FM31	*Sphaeranthus indicus*	Flower	*rbcL*	OR381719
FM31	*Sphaeranthus indicus*	Flower	*psbA-trnH*	OR381758
FM31	*Sphaeranthus indicus*	Flower	*ITS2*	OR400839
FM31	*Sphaeranthus indicus*	Flower	Mini	OR400887
FM33	*Lobularia maritima*	Leaf	*rbcL*	OR381711
FM33	*Lobularia maritima*	Leaf	*psbA-trnH*	OR381748
FM33	*Lobularia maritima*	Flower	*psbA-trnH*	OR381749
FM33	*Lobularia maritima*	Leaf	*ITS2*	OR400830
FM33	*Lobularia maritima*	Flower	*ITS2*	OR400831
FM33	*Lobularia maritima*	Leaf	Mini	OR400869
FM34	*Tropaeolum majus*	Leaf	*rbcL*	OR381726
FM34	*Tropaeolum majus*	Leaf	*psbA-trnH*	OR381766
FM34	*Tropaeolum majus*	Leaf	*ITS2*	OR400847
FM34	*Tropaeolum majus*	Flower	*ITS2*	OR400848
FM34	*Tropaeolum majus*	Leaf	Mini	OR400877
FM35	*Taraxacum officinale*	Leaf	*rbcL*	OR381723
FM35	*Taraxacum officinale*	Leaf	*psbA-trnH*	OR381763
FM35	*Taraxacum officinale*	Leaf	*ITS2*	OR400844
FM35	*Taraxacum officinale*	Leaf	Mini	OR400875
FM35	*Taraxacum officinale*	Flower	Mini	OR400876

Table S2. List of PCR primers used.

Target species	Region	Anneal	Forward primer	Reverse primer	Reference*
Universal plant barcode	*rbcL*	55	ATGTCACCACAAACAGAGACTAAAGC	GTAAAATCAAGTCCACCRCG	[80–83] in [79]
Universal plant barcode	*trnH-psbA*	55	GTTATGCATGAACGTAATGCTC	CGCGCATGGTGGATTCACAATCC	[84, 85] in [79]
Universal plant barcode	*ITS2*	55	ATGCGATACTTGGTGTGAAT	TCCTCCGCTTATTGATATGC	[86, 87] in [79]
Achillea millefolium	*ITS1*	54	TGGACCTGGTAAATGTGTCAT	GATGCGTGAGCCGAGATA	(this study)
Agastache foeniculum	*ITS2*	60	ATTAGGCCGAGGGCACGTC	CGACGGCGCGAGATTGATG	(this study)
Amaranthus hypochondriacus	*ITS2*	58	GATGGTCTCCCATGCCTCAC	GCAACGCTCTAGGGTCCTC	(this study)
Antirrhinum majus	*ITS2*	60	ATTAGGCCGAGGGCACGTC	ACGGAGCCAGTCACGACAAC	(this study)
Bougainvillea glabra	*ITS2*	55	CTCCCTCATCCACCCTTTATG	CATGCGAGCTGCAAATGAC	(this study)
Brassica oleracea	*ITS2*	56	TGTGAATTACAGAATCCCGTGAAC	GACGCTCCTGGCATCCTTAG	(this study)
Calendula officinalis	*ITS1*	55	CAGCAGAATGACCCGTGAAC	CGAGCCCTTCTTAGGTTTAGTATT	(this study)
Chenopodium album	*trnH-psbA*	53	AACTTCCCTCTAGACTTAGCC	GTAAAGGTAAAGGAGCAATACCC	(this study)
Chrysanthemum × morifolium	*trnH-psbA*	50	ATTTAGTACTATTTGCCTTACACAG	TTGAATTGGAAATCAAACTTCATAA	(this study)
Hibiscus rosa-sinensis	*trnH-psbA*	54	AAGGAGCAATACCAATTTCTTGTTC	GGGTAGGTAATAGGGTAAAGGG	(this study)
Hibiscus sabdariffa	*ITS1*	55	GAATTACGCCAAGGAATCGGAAT	TCACACCAAGTATCGCATTTCG	(this study)
Lantana camara	*ITS2*	56	GTTGGCCCAAATGCGATCC	TTAAACTCAGCGGGTAATCCC	(this study)
Lobularia maritima	*ITS2*	56	CTGCCTGGGTGTCACAAATC	TATATGACGAGGCTTGTATTCACCAC	(this study)
Pelargonium zonale	*ITS2*	60	CCCTACCACCGAATGCCAGTTT	CCCGCAAGGAGGAGAGGAAG	(this study)
Rosa chinensis	*ITS2*	60	GTTGCGCCCGAAGCCATTAG	CGACACGCATTGTTTAAGAAAGCACTC	(this study)
Ruta graveolens	*ITS2*	57	CTGGGTGTCACGCATCATTG	ACAGGAACTCGAGAGGCTTTG	(this study)
Sinapis arvensis	*ITS1*	54	GGTCTCTTAGCCGATTCCGT	GCAGCACTACTTCCGAACAA	(this study)
Sphaeranthus indicus	*ITS1*	55	CCCGAGAACCTGTACAAACTG	GAACGTGGTGACAAGGCATA	(this study)
Tagetes erecta	*ITS2*	55	CCACACCATGACTCCCTCTT	CCACTAGTCGTGCATCCATC	(this study)
Tanacetum vulgare	*trnH-psbA*	51	GCAATAGCTTTCCTCTTGTT	TGGAATAGAATATACAAATACAAACCT	(this study)
Taraxacum officinale	*ITS2*	57	AGGGTAGTCGTGGTGATTGG	GTCCGAAGCATCCGTCCTA	(this study)
Trifolium pratense	*ITS2*	55	GCAGAATCCCGTGAACCAT	GCACACTCTACCAAGGTCTC	(this study)
Tropaeolum majus	*ITS2*	58	TCGTATCGCCACTCTCTTTCTTTGAATATC	GGTCTGACCAAGGAGCACAC	(this study)

* see main article for citations

79. Fazekas, A.J.; Kuzmina, M.L.; Newmaster, S.G.; Hollingsworth, P.M. DNA barcoding methods for land plants. In *DNA Barcodes: Methods and Protocols*; Kress, W.J., Erickson, D.L., Eds.; Humana Press: Totowa, NJ, USA, 2012; pp. 223–252.
80. Soltis, P.S.; Soltis, D.E.; Smiley, C.J. An *rbcL* sequence from a Miocene *Taxodium* (Bald Cypress). *Proc. Natl. Acad. Sci. USA* **1992**, *89*, 449–451. https://doi.org/10.1073/pnas.89.1.449.
81. Fofana, B.; Harvengt, L.; Baudoin, J.P.; Jardin, P.D. New primers for the polymerase chain amplification of cpDNA intergenic spacers in *Phaseolus* phylogeny. *Belg. J. Bot.* **1997**, *129*, 118–122.
82. Levin, R.A.; Wagner, W.L.; Hoch, P.C.; Nepokroeff, M.; Pires, J.C.; Zimmer, E.A.; Sytsma, K.J. Family-level relationships of Onagraceae Based on chloroplast *rbcL* and *ndhF* data. *Am. J. Bot.* **2003**, *90*, 107–115. https://doi.org/10.3732/ajb.90.1.107.

83. Kress, W.J.; Erickson, D.L.; Jones, F.A.; Swenson, N.G.; Perez, R.; Sanjur, O.; Bermingham, E. Plant DNA barcodes and a community phylogeny of a tropical forest dynamics plot in Panama. *Proc. Natl. Acad. Sci. USA* **2009**, *106*, 18621–18626. https://doi.org/10.1073/pnas.0909820106.
84. Sang, T.; Crawford, D.J.; Stuessy, T.F. Chloroplast DNA phylogeny, reticulate evolution, and biogeography of *Paeonia* (Paeoniaceae). *Am. J. Bot.* **1997**, *84*, 1120–1136. https://doi.org/10.2307/2446155.
85. Tate, J.A.; Simpson, B.B. Paraphyly of *Tarasa* (Malvaceae) and diverse origins of the polyploid species. *Syst. Bot.* **2003**, *28*, 723–737.
86. Chen, S.; Yao, H.; Han, J.; Liu, C.; Song, J.; Shi, L.; Zhu, Y.; Ma, X.; Gao, T.; Pang, X.; et al. Validation of the *ITS2* region as a novel DNA barcode for identifying medicinal plant species. *PLoS ONE* **2010**, *5*, e8613. https://doi.org/10.1371/journal.pone.0008613.
87. White, T.J.; Bruns, T.; Lee, S.; Taylor, J. Amplification and direct sequencing of fungal ribosomal RNA genes for phylogenetics. In *PCR Protocols: A Guide to Methods and Applications*; Academic Press: New York, NY, USA; pp. 315–322.
